# SAAP-148 and halicin exhibit synergistic antimicrobial activity against antimicrobial-resistant bacteria in skin but not airway epithelial culture models

**DOI:** 10.1093/jacamr/dlaf050

**Published:** 2025-04-11

**Authors:** Patrick R Lennard, Pieter S Hiemstra, Julia R Dorin, Peter H Nibbering

**Affiliations:** PulmoScience Laboratory, Department of Pulmonology, Leiden University Medical Center, Leiden, The Netherlands; Laboratory of Infectious Diseases, Leiden University Center of Infectious Diseases, Leiden University Medical Center, Leiden, The Netherlands; Centre for Inflammation Research, Institute for Regeneration and Repair, University of Edinburgh, Edinburgh, UK; Institute of Immunology and Infection Research, School of Biological Sciences, University of Edinburgh, Edinburgh, UK; PulmoScience Laboratory, Department of Pulmonology, Leiden University Medical Center, Leiden, The Netherlands; Centre for Inflammation Research, Institute for Regeneration and Repair, University of Edinburgh, Edinburgh, UK; Laboratory of Infectious Diseases, Leiden University Center of Infectious Diseases, Leiden University Medical Center, Leiden, The Netherlands

## Abstract

**Background:**

The escalating global threat of antimicrobial resistance (AMR) necessitates the development of novel antimicrobial agents, innovative strategies, and representative infection models to combat AMR bacterial infections. Host defence peptides (HDPs) and their derivatives have been proposed as complements to conventional antibiotics due to their antibacterial activity and modulation of the immune response.

**Objectives:**

This study investigated the novel use of the HDP-derived synthetic antibacterial and anti-biofilm peptide (SAAP)-148 as a pretreatment in epithelial tissue models to prevent colonization by AMR bacteria. The combined activities of SAAP-148 pretreatment with post-infection halicin to treat infections were also explored.

**Methods:**

Employing cultured human skin equivalents (HSEs) and primary bronchial epithelial cells (PBECs) as models of tissue infection, we examined the prophylactic and therapeutic effects of SAAP-148, both singularly and in combination with the repurposed antibiotic halicin, against AMR bacteria. We additionally interrogated the response of HSE and PBEC cultures to SAAP-148 treatment via confocal microscopy and quantitative PCR of native HDPs and inflammatory cytokine genes.

**Results:**

Our findings demonstrated that pretreatment with SAAP-148 significantly reduces colonization of HSEs and PBECs by AMR *Staphylococcus aureus* and *Pseudomonas aeruginosa*. Confocal microscopy revealed differential uptake and localization of SAAP-148 in these tissues, correlating with its distinct activity in these tissues. SAAP-148 exposure temporarily increased expression of the HDPs cathelicidin (*CAMP*) and β-defensin 1 (*DEFB1*), and the cytokine IL-8 (*CXCL8*), which did not correlate with the transient antibacterial activity observed. Sequential treatment with SAAP-148 prior to infection with AMR *S. aureus* and post-infection halicin treatment demonstrated synergistic activity in HSEs, whereas this combined activity was indifferent in PBEC cultures.

**Conclusions:**

These results support SAAP-148 as a candidate for pre-infection prophylaxis and synergistic antibiotic therapy with halicin in skin, broadening the potential of both agents to address AMR bacterial infection.

## Introduction

The global burden of bacterial antimicrobial resistance (AMR) accounts for approximately 1.3 million deaths annually,^1^ and is predicted to surpass 10 million deaths annually by 2050.^2^ To combat this, novel antibacterial agents must be developed to complement and reinvigorate current therapies. Given the dwindling development of antimicrobial agents,^3^ host defence peptides (HDPs) are proposed as alternative agents, combining broad-spectrum antimicrobial (antibacterial, antifungal, antiviral, anti-biofilm and toxin-neutralizing) capabilities with modulation of host immune defence and repair mechanisms,^4,5^ largely due to their direct targeting of the bacterial membrane.^6^

LL-37, the active peptide of human cathelicidin, is the most studied of the human HDPs, exhibiting direct antibacterial activity in addition to immunomodulatory effects.^7,8^ A library of synthetic antibacterial and anti-biofilm peptides (SAAPs) was developed from LL-37 homologue consensus sequences, and SAAP-148 has emerged as a leading candidate due to its enhanced microbicidal and anti-biofilm activity,^9^ and facilitation of wound repair.^10^ Despite its cytotoxicity and haemolysis at functional concentrations limiting its clinical advancement,^11^ SAAP-148 may potentiate and synergize with other antibiotics, and optimize the immune response against AMR infections similar to other HDP derivatives.^12^ Halicin was recently identified and repurposed to combat AMR bacteria with the aid of deep learning technology,^13^ and has proven to have potent synergistic activities with SAAP-148 against AMR strains of *Staphylococcus aureus*, *Escherichia coli* and *Klebsiella pneumoniae*,^14,15^ and with doxycycline against *Enterococcus faecalis* and *Enterococcus faecium*.^16^ Halicin drives bacterial death through disruption of the transmembrane electrochemical gradient, as opposed to the discrete bacterial targets of conventional antibiotics. Together with SAAP-148 and other HDPs, halicin provides new means to combat AMR bacteria in combination treatment and alternative models of antibiotic therapy.

Here, we assessed the effect of SAAP-148 pretreatment on colonization of human skin equivalents (HSEs) and primary bronchial epithelial cells (PBECs) by AMR *S. aureus* and *Pseudomonas aeruginosa* strains. Thereafter, we investigated the interactions between SAAP-148 and cells in HSEs and PBECs, and its stimulation of HDP and inflammatory cytokine expression in these cultures. Given the peptide’s efficacy in combination with the novel antibiotic halicin,^14,15^ SAAP-148 pretreatment of HSE and PBEC cultures was combined with post-infection halicin treatment to examine if such synergy would be retained in sequential therapy. Together, our results demonstrate the potential of SAAP-148 alone and in combination with halicin for the prevention and possible treatment of skin infection by AMR bacteria.

## Materials and methods

### Ethics

This study was conducted in accordance with the Declaration of Helsinki. Ethical approval was not required for studies involving human lung tissue due to patient donor enrolment in the no-objection system for coded anonymous further use of such tissue (www.coreon.org). As of 1 September 2022, patients are enrolled in the biobank using active informed consent, in accordance with the local regulations of the Leiden University Medical Center (LUMC) biobank with approval by the institutional medical ethical committee (B20.042/Ab/ab and B20.042/Kb/kb). All human samples used were acquired as a by-product of routine care.

### Generation of HSE and PBEC cultures

HSEs were generated from the Ker-CT cell line of immortalized keratinocytes derived from infant foreskin (ATCC^®^ CRL-4048™), cultured according to a previous method.^14^ Briefly, cells were seeded onto 0.4 µm transwell inserts (Greiner) in 12-well cell culture plates, supplemented with DermaLife K medium (Lifeline Cell Technology), and incubated at 37°C and 5% CO_2_. As cells became confluent across transwell inserts, medium was replaced with K0:CNT medium supplemented with 1:1:1 lipid mixture as described previously.^14^ After overnight incubation, apical medium was removed to air-expose cultures. HSEs were cultured for a further 10 days, with basal culture medium replenished with fresh K0:CNT medium with 2:1:1 lipid mixture every 2–3 days. PBECs were cultured and differentiated at the air–liquid interface as previously described.^17^ In short, bronchial epithelial cells were isolated from macroscopically normal, tumour-free bronchus rings obtained from biobank-enrolled patients undergoing resection surgery for lung cancer at the LUMC. Bronchial cells from five donors were seeded as 150 000 cells (30 000 per donor) per transwell insert in 500 µL of complete BD composite medium^17^ in wells of the same medium. Two days after cell layers became confluent, apical medium was removed to air-expose cultures. PBECs were cultured for a further 2 weeks to ensure appropriate differentiation. Alternating donor mixes were used for every independent experimental replicate, with duplicate technical replicates prepared for each experimental condition. Prior to treatment, excess mucus was removed by washing PBEC cultures with PBS (500 µL) over a 10 min incubation. Culture media were replenished with their respective antibiotic-free medium at least 1 day prior to experimentation. All cultures and incubations were conducted at 37°C and 5% CO_2_.

### Antibacterial agents and application

SAAP-148 (acetyl-LKRVWKRVFKLLKRYWRQLKKPVR-amide) and FITC-conjugated SAAP-148 were synthesized by Fmoc chemistry, purified by HPLC, and their identity confirmed by MS as previously described.^9,18^ SAAP-148 was dissolved in PBS to a concentration of 16.75 g/L, and diluted to working concentrations ≤335 mg/L in PBS, to maintain its conformation-dependent activity similar to LL-37.^19^ HSEs and PBECs were incubated with peptide suspension (200 μL) for 24, 6 or 1 h prior to inoculation. Halicin (Tocris Bioscience, UK) was dissolved in DMSO to a concentration of 13.5 g/L and diluted in PBS to ≤54 mg/L.

### Bacterial culture, inoculation of HSE and PBEC cultures, and microbiological determination of antibacterial efficacy

MRSA strain LUH14616 [NCCB 100829; MDR clinical isolate (aminoglycoside-, fluoroquinolone-, penicillin- and tetracycline-resistant^9,20^)] and *P. aeruginosa* strain LUH15103 [MDR (aminoglycoside-, carbapenem-, cephalosporin-, fluoroquinolone-, monobactam- and penicillin-resistant) clinical isolate] were streaked on blood agar plates (bioMérieux, France) and incubated overnight. Bacteria were cultured in tryptic soy broth (TSB; Oxoid, UK) at 200 rpm for 2.5 h, centrifuged at 3600 rpm for 10 min, resuspended in PBS, centrifuged again and resuspended as 15 000 cfu/mL in respective culture medium without antibiotics, as estimated by optical density at 600 nm. HSE and PBEC cultures were inoculated with ∼300 cfu in 20 μL and then incubated for 4 h. For post-infection treatment, apical surfaces were washed with PBS 1 h after inoculation, and halicin was applied for 3 h.

The non-adherent cell-free (CF) bacterial fraction was collected by washing transwell insert cultures with PBS (180 µL) after inoculation and/or treatment. The cell-adherent and biofilm-established (CA) bacterial fraction was obtained by excision of insert membranes from inserts and disruption with glass beads using a Precellys 24 tissue homogenizer (Bertin Instruments, France) at 5000 rpm for 3 × 10 s. Ten-fold serial dilutions of CF and CA bacterial fractions and control inocula were prepared, and 20 μL of each dilution dispensed onto Mueller–Hinton agar plates (Oxoid). Plates were incubated overnight and colonies counted. Plates without bacterial growth were incubated again overnight to confirm complete elimination. MICs were determined as the lowest concentration of agent at which there is no visible growth of bacteria.^21^

### Confocal imaging of SAAP-148 interactions in HSE and PBEC cultures

HSEs and PBECs were exposed to 84 mg/L of SAAP-148 (spiked 1:100 with FITC-SAAP-148) in PBS or as control to PBS for 1, 6 or 24 h, after which the cultures were fixed in 1% (v/v) paraformaldehyde (PFA; Biotium, USA) for 1 h at 4°C. Spiking of SAAP-148 treatment with FITC-labelled peptide was performed to eliminate excessive tissue staining. Insert cultures were washed and stored in PBS at 4°C until staining. HSE and PBEC cultures were blocked with PBS containing 1% (w/v) BSA and 0.3% (v/v) Triton™ X-100 (PBS with Triton^TM^ detergent; PBT) for 15 min at room temperature. Insert membranes were excised, washed once in PBS, and incubated with goat anti-human EpCAM antibody (R&D Systems, USA; 10 mg/L) in PBT overnight at 4°C. Membranes were washed thrice in PBS, then incubated with rabbit anti-goat antibody with Alexa Fluor™ 594 (Invitrogen, USA) and DAPI (Invitrogen), diluted 1:200 and 1:100 respectively in PBT, for 2 h at 4°C. Membranes were washed thrice in PBS, thrice more in deionized water, then placed on a glass slide, and treated with ProLong Diamond Antifade mountant (Invitrogen). Z-stacks were imaged with 0.22 µm intervals using a Leica LSM900 Airyscan inverted confocal microscope (Leica Microsystems, Germany) with a 40 ×  oil-immersion objective lens. Images and orthogonal projections were generated and deconvoluted with ZEN 3.11 blue edition software (Zeiss, Germany). Quantitation of cells and individual cellular fluorescence intensities was performed with CellProfiler version 4.2.8 (Broad Institute, USA).^22^

### Determination of gene expression levels

HSE and PBEC cultures were treated with 200 μL of 84 mg/L SAAP-148 or PBS for 1, 6, 12 or 24 h. Total RNA from homogenized HSEs and PBECs was extracted using TRIsure (Bioline, UK) after bead-beating in a Precellys 24 tissue homogenizer. cDNA synthesis was performed on 500 ng of total RNA per sample using the GoScript™ Reverse Transcription System (Promega, USA) according to the manufacturer's instructions. Quantitative PCR (qPCR) was performed using a QuantStudio™ 6 Flex Real-Time PCR System (Applied Biosystems, USA), with sample volumes of 25 μL, comprising 1× GoTaq^®^ qPCR Master Mix (Promega), 10 pmol each primer (Table 1), and 5 μL of 10× diluted cDNA. PCR conditions consisted of: denaturation at 95°C for 2 min; denaturation at 95°C for 15 s, then annealing and extension at 60°C for 1 min, for 40 cycles; and denaturation at 95°C for 15 s, annealing at 56°C for 1 min, followed by melt curve analysis from 56°C to 95°C in increments of 0.2°C. Samples were performed in duplicate and analysed using QuantStudio™ Real-Time PCR Software (Applied Biosystems). Data are presented as relative fold-change of expression after standardization to levels of β_2_-microglobulin (B2M) and GAPDH. These genes were selected from a panel of candidates using the RefFinder tool, which integrates available algorithms for reference gene comparison and ranking.^23^

**Table 1.** Primer sequences used for reverse transcription quantitative PCR

Target sequence	Primer sequence	
	Forward	Reverse
B2M (*B2M*)	TGCTGTCTCCATGTTTGATGTATCT	TCTCTGCTCCCCACCTCTAAGT
GAPDH (*GAPDH*)	AAGGTCGGAGTCAACGGATTT	ACCAGAGTTAAAAGCAGCCCTG
LL-37 (*CAMP*)	ATTTCTCAGAGCCCAGAAGC	CGGAATCTTGTACCCAGGAC
IL-6 (*IL6*)	GGTACATCCTCGACGGCATCT	GTGCCTCTTTGCTGCTTTCAC
IL-8 *(CXCL8)*	GCCAGGAAGAAACCACCGGAAGG	GGCTGCCAAGAGAGCCACGG
hBD-1 *(DEFB1)*	TGAGTGTTGCCTGCCAGT	TCTTCTGGTCACTCCCAG
hBD-2 *(DEFB4A/B)*	TGATGCCTCTTCCAGGTGTTT	GGATGACATATGGCTCCACTCTTA
hBD-3 *(DEFB103A)*	TTATTGCAGAGTCAGAGGCGG	CGAGCACTTGCCGATCTGTT

### Checkerboard assay

HSE and PBEC cultures were treated with SAAP-148 for 1 h prior to infection. The apical surface was washed with PBS and ∼300 cfu of MRSA strain LUH14616 applied in 20 µL of PBS. After 1 h incubation, insert cultures were washed, treated with halicin for 3 h, and both CF and CA bacterial suspensions collected. Serial dilution, plating and overnight incubation of suspensions were performed as above to determine agent MICs. The FIC index (FICi) scores for CF and CA fractions were calculated from the MICs of individual agents (MIC_SAAP-148, alone_, MIC_halicin, alone_) and their reduced MICs in combination with the other agent (MIC_SAAP-148, combination_, MIC_halicin, combination_). The latter figure divided by the former also yields an FIC that indicates the effect of the alternative agent on the principal (FIC_H→S_, FIC_S→H_). These figures are derived using the following formula:


$$\mathrm{FICi}\;=\;\mathrm{FI}C_{H\to S}+\mathrm{FI}C_{S\to H}\;=\;\;\frac{\mathrm{MI}C_{\mathrm{SAAP}-148,\;\;\mathrm{combination}}}{\mathrm{MI}C_{\mathrm{SAAP}-148,\;\mathrm{alone}}}+\frac{\mathrm{MI}C_{\mathrm{halicin},\;\;\mathrm{combination}}}{\mathrm{MI}C_{\mathrm{halicin},\;\mathrm{alone}}}$$

Interactions were determined as: FICi ≤0.5 indicates synergism; 0.5 < FICi < 4 indicates no effect; and FICi >4 indicates antagonism between agents.^24,25^ The influence of halicin on SAAP-148 (FIC_H→S_) is determined as: FIC_H→S_ ≤ 0.25 indicates that halicin promotes the activity of SAAP-148; 0.25 < FIC_H→S_ < 2 indicates no effect; and FIC_H→S_ > 2 indicates antagonism between agents.^26^ The FIC_S→H_ indicates the reciprocal influence of SAAP-148 upon halicin’s activity.

### Statistics

Differences between two or more treatment groups for antimicrobial efficacy were evaluated using analysis of variance (ANOVA) with treatment and experimental replicate as factors if data were normally distributed according to an Anderson–Darling normality test. In case of non-normal distribution, data were evaluated by the non-parametric Kruskal–Wallis test. Assessment between specific groups was conducted using the post-hoc Dunn’s multiple comparisons test. Data from qPCR were assessed by two-way ANOVA with Šidák’s correction for multiple comparisons. All statistical testing was performed in GraphPad Prism software version 9.0 (Graph Pad Software, USA). Differences were considered statistically significant when *P* < 0.05.

## Results

### Exposure of HSEs and PBECs to SAAP-148 prevents colonization by AMR bacteria

**Figure 1.**
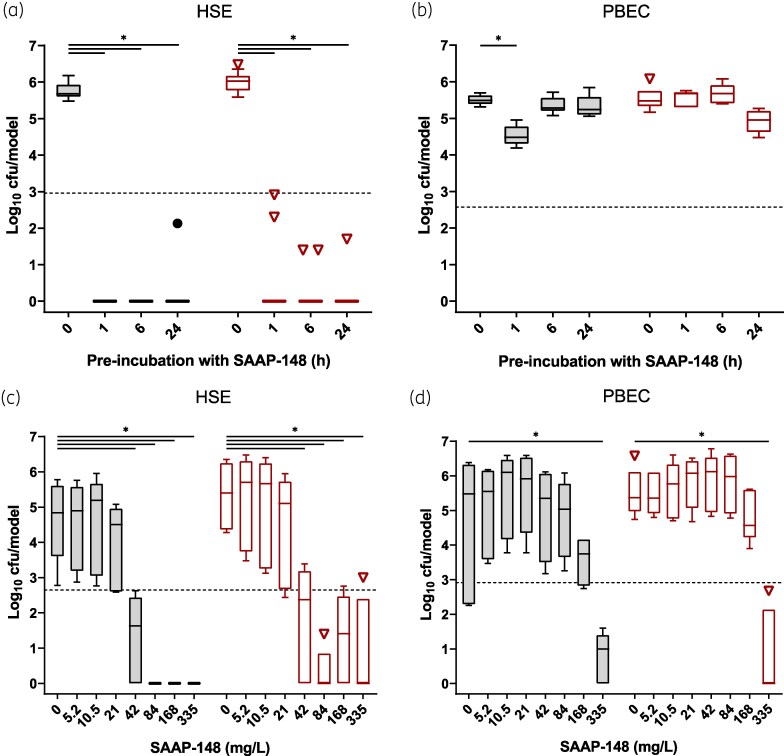
Effect of pre-exposure of HSEs and PBECs to SAAP-148 on colonization by MRSA. Bacterial counts from cell-free (CF; grey) and cell-associated (CA; red) fractions of HSE and PBEC cultures after pretreatment with SAAP-148 and infection with MRSA LUH14616. HSE (a) and PBEC (b) cultures were pretreated with 84 mg/L SAAP-148 for 1, 6 or 24 h, exposed to MRSA LUH14616 for 4 h, and bacterial fractions retrieved and assessed microbiologically. Next, HSE (c) and PBEC (d) cultures were pretreated for 1 h with increasing concentrations of SAAP-148, then subsequently infected with MRSA LUH14616 for 4 h, and bacterial fractions retrieved. Results are expressed as box-and-whisker plots with Tukey range, with outliers plotted individually (black dots for CF, red triangles for CA). Data are from three independent experiments consisting of triplicate (HSE) or duplicate (PBEC) technical replicates. Statistical differences between control and SAAP-148 pretreated models are depicted (*P ≤ 0.05) as calculated by one-way ANOVA or Kruskal–Wallis tests with Dunn’s adjustment for multiple comparisons. Dashed lines indicate the LC_99.9_, i.e. the threshold for a 99.9% reduction in bacterial counts compared with the control.

**Figure 2.**
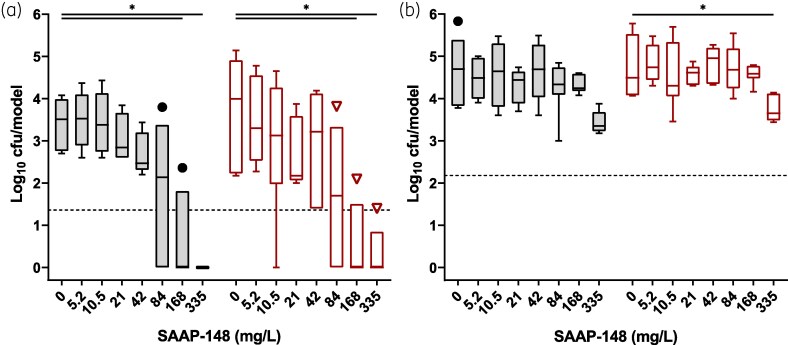
Effect of pre-exposure of HSEs and PBECs to SAAP-148 on colonization by AMR *P. aeruginosa*. Bacterial counts from cell-free (CF; grey) and cell-associated (CA; red) fractions of HSE and PBEC cultures after pretreatment with SAAP-148 and infection with *P. aeruginosa* LUH15103. HSE (a) and PBEC (b) cultures were pretreated with increasing concentrations of SAAP-148 for 1 h, exposed to *P. aeruginosa* LUH15103 for 4 h, and bacterial fractions retrieved and assessed microbiologically. Results are expressed as box-and-whisker plots of the Tukey range, with outliers plotted individually (black dots for CF, red triangles for CA). Data are from three independent experiments consisting of triplicate (HSE) or duplicate (PBEC) technical replicates. Statistical differences between control and SAAP-148 pretreated models are depicted (*P ≤ 0.05) as calculated by one-way ANOVA or Kruskal–Wallis tests with Dunn’s adjustment for multiple comparisons. Dashed lines indicate the LC_99.9_, i.e. the threshold for a 99.9% reduction in bacterial counts compared to the control.

To investigate if SAAP-148 can protect epithelia from bacterial colonization, we exposed HSEs and PBECs to 84 mg/L SAAP-148 for 1, 6 or 24 h prior to a 4 h infection with MRSA LUH14616. Bacterial counts of CF and CA fractions revealed that pretreatment of HSEs with SAAP-148 significantly (*P* < 0.001) prevented colonization at all time points (Figure 1a). Pretreatment of PBECs with SAAP-148 demonstrated limited protection against bacterial colonization (Figure 1b), with significant reduction occurring only after 1 h pretreatment in the CF fraction (*P* = 0.01). Bacterial counts in neither fraction dropped below the threshold for eliminating 99.9% of bacteria (LC_99.9_). To validate the protective efficacy of SAAP-148, HSEs and PBECs were exposed to a range of SAAP-148 concentrations for 1 h prior to a 4 h infection with MRSA. Pretreatment of HSEs with ≥42 mg/L of SAAP-148 resulted in significantly reduced CF (*P* < 0.04) and CA (*P* < 0.03) bacterial counts (Figure 1c). Pretreatment of PBECs with 335 mg/L of SAAP-148 also significantly (*P* < 0.01) reduced both CF and CA bacterial counts (Figure 1d). To assess this protective potential against a Gram-negative bacterium, HSEs and PBECs were exposed to a range of SAAP-148 concentrations for 1 h and then inoculated with the *P*. *aeruginosa* strain LUH15103. After 4 h, bacterial counts in the CF and CA suspensions in HSEs treated with ≥168 mg/L SAAP-148 were significantly (*P* < 0.02) reduced (Figure 2a), whereas experiments with PBECs required pretreatment with 335 mg/L SAAP-148 to significantly (*P* < 0.03) reduce CA bacterial counts (Figure 2b), again failing to drop below the LC_99.9_. These results indicate that the preventative effects of SAAP-148 on HSE colonization by AMR bacteria were more pronounced than on colonization of PBECs.

### Tissue retention of FITC-SAAP-148 in HSEs and PBECs

**Figure 3.**
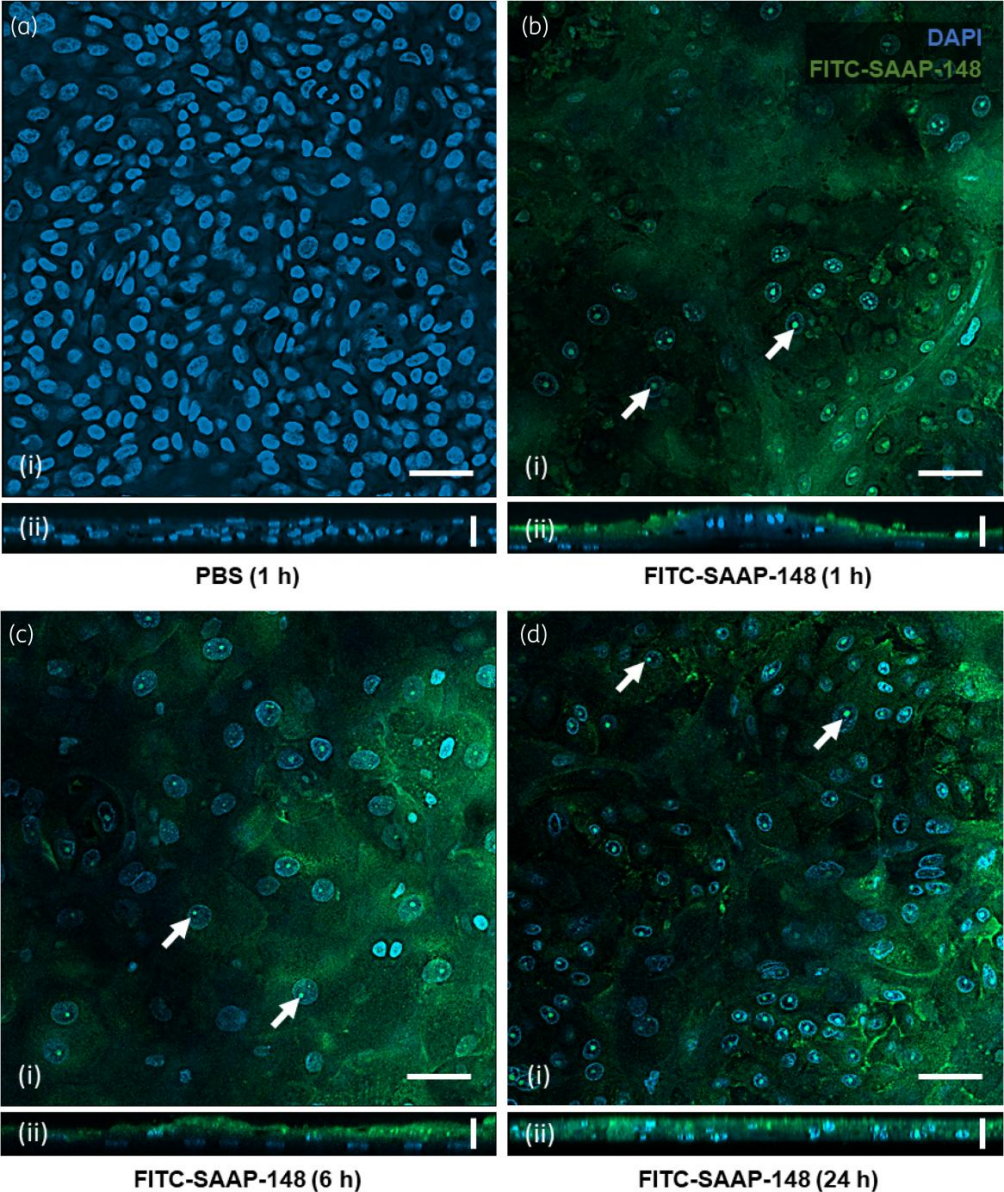
Confocal microscopy of HSEs exposed to FITC-SAAP-148. HSEs were exposed to either PBS for 1 h (a) or FITC-SAAP-148 (green) for 1 h (b), 6 h (c) or 24 h (d), washed, and stained with DAPI (blue, nucleus). Apical layer images (i) and orthogonal z-stack projections (ii) are shown. FITC-SAAP-148 localizes largely to apical layer cell membranes as apparent from orthogonal projections, and forms puncta localized to cell nuclei (white arrows). Images were taken with 40 ×  oil immersion lens and are shown as representative images of three inspection fields surveyed per sample, from three independent experiments each performed in duplicate. Scale bars represent 50 µm (i) and 20 µm (ii).

**Figure 4.**
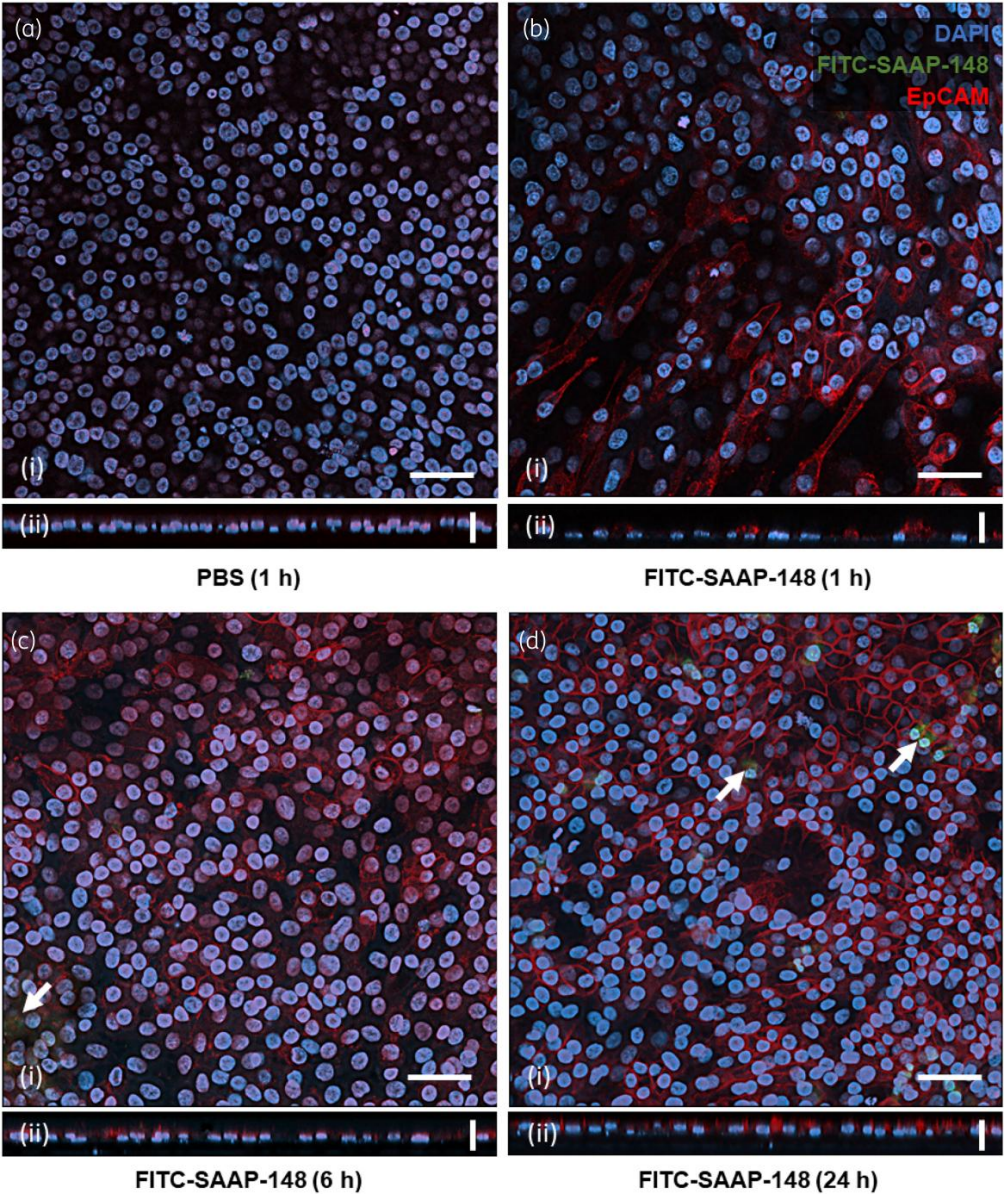
Confocal microscopy of PBECs treated with FITC-SAAP-148. PBEC cultures were exposed to either PBS for 1 h (a) or FITC-SAAP-148 (green) for 1 h (b), 6 h (c) or 24 h (d), washed, incubated with goat anti-human EpCAM antibody overnight, then washed and stained with rabbit anti-goat IgG-Alexa Fluor™ 594 (red, cell membrane) and DAPI (blue, nucleus) for 2 h prior to washing and mounting. Apical layer images (i) and orthogonal z-stack projections (ii) are shown. FITC-SAAP-148 staining was not apparent except for speckling at later time points (white arrows). Images were taken with 40 ×  oil immersion lens and are shown as representative images of three inspection fields surveyed per sample, from three independent experiments, each performed in duplicate. Scale bars represent 50 µm (i) and 20 µm (ii).

To understand the different antimicrobial activities of SAAP-148 observed between tissue cultures, HSE and PBEC cultures were exposed to 84 mg/L SAAP-148, spiked 1:100 with FITC-SAAP-148, for periods of 1, 6, or 24 h. Cultures grown on transwell inserts were stained with goat anti-human EpCAM antibody overnight, and further stained with both secondary rabbit anti-goat IgG-Alexa Fluor™ 594 and DAPI for 2 h before mounting. Localization of the peptide was investigated by confocal laser scanning microscopy, revealing a haze of FITC-SAAP-148 fluorescence on the surface of HSEs (Figure 3) and within cells, except for untreated cultures (Figure 3a). FITC-SAAP-148 staining was coincident with nuclei staining up to 24 h after application (Figure 3b–d). In PBECs, however, FITC-SAAP-148 was less evident with marginal peptide puncta formation in the cells (Figure 4). Quantitative analysis of cellular FITC-SAAP-148 fluorescence (Figure S1, available as Supplementary data at *JAC-AMR* Online) validated its presence in HSEs up to 24 h after application (*P* < 0.02), whereas in PBEC cultures the fluorescence did not increase from untreated samples and reduced at 24 h (*P* < 0.01). The number of nucleated cells did not change (Figure S2), across cultures or time points of treatment (*P* > 0.32). Together, these data suggest that SAAP-148 sticks to the surface and is internalized by HSEs, whereas only minor amounts of SAAP-148 are associated with epithelial cells in PBECs.

### HDP and inflammatory cytokine expression in HSEs and PBECs after SAAP-148 exposure

**Figure 5.**
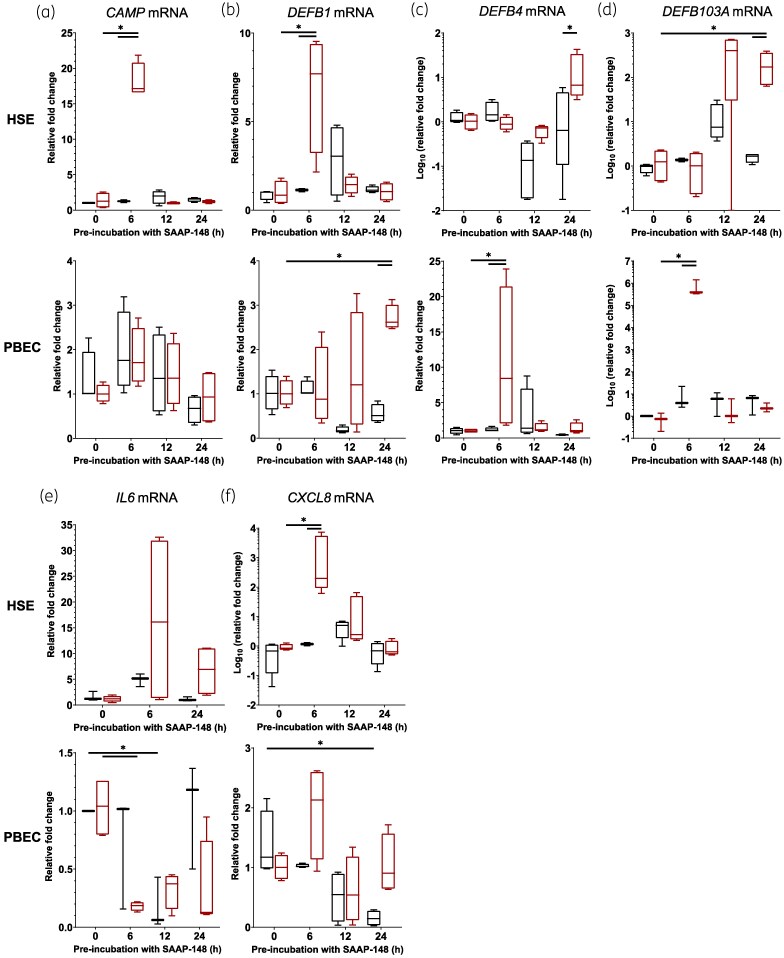
Expression of host defence peptides and inflammatory cytokines by HSEs and PBECs in response to SAAP-148. HSEs and PBECs were exposed to SAAP-148 (red) or—as control—to PBS (black) for up to 24 h, and then RNA was extracted from the models for assessment of mRNA levels of LL-37 (a, *CAMP*), hBD-1 (b, *DEFB1*), hBD-2 (c, *DEFB4*), hBD-3 (d, *DEFB103A*), IL-6 (e, *IL-6*) and IL-8 (f, *CXCL8*) by qPCR. Results are displayed as mean fold changes in mRNA expression levels relative to the geometric mean of housekeeping genes  ± standard errors of the means from four independent experiments, each performed as duplicate technical replicates. Significant differences between treatments and time points from controls are depicted (*P ≤ 0.05) as calculated by two-way ANOVA with Šidák’s correction for multiple comparisons.

Since we observed that SAAP-148 appears to interact with epithelial cell membranes and may accumulate in the nucleus, we considered the possibility that SAAP-148 can modulate gene expression, similar to LL-37, the HDP on which the development of SAAP-148 was based. HDP stimulation of both keratinocytes and bronchial epithelial cells has shown induction of expression of both other HDP genes and inflammatory genes.^27–30^ To this end, we explored the capacity of SAAP-148 to induce the expression of LL-37 (*CAMP*), other potent HDPs—the β-defensins (*DEFB1*, *DEFB4*, *DEFB103A*)—and the inflammatory cytokines IL-6 (*IL6*) and IL-8 (*CXCL8*), respectively indicating the stimulation of endogenous HDP expression and inflammatory signalling. Results revealed that expression of *CAMP* by cells in HSEs, but not PBECs, was significantly (*P* < 0.001) increased at 6 h of exposure to SAAP-148, after which the levels subsided, returning to control levels at 12 h after peptide exposure (Figure 5a); *DEFB1* [human β-defensin 1 (hBD-1)] expression by cells in HSEs was significantly increased transiently at 6 h upon SAAP-148 exposure (*P *< 0.001), gradually decreasing to control levels at 24 h, whereas expression levels in PBECs increased incrementally across 24 h of exposure to SAAP-148, being significantly (*P* = 0.02) different from control samples only at 24 h after SAAP-148 application (Figure 5b). Expression of *DEFB4* [human β-defensin 2 (hBD-2)] by cells in SAAP-148–treated HSEs was significantly (*P* = 0.03) greater than the control after 24 h, but this was not greater than baseline expression (*P* = 0.301). The same expression was transiently increased by cells in PBECs at 6 h after peptide application (Figure 5c, *P* = 0.05). Similarly, *DEFB103A* [human β-defensin 3 (hBD-3)] expression by cells in PBECs was significantly (*P* < 0.001) increased at 6 h of exposure to SAAP-148 before decreasing to control levels, whereas expression of this gene by cells in HSEs increased (>100-fold; *P* < 0.02) at 24 h after SAAP-148 application (Figure 5d). Expression of the inflammatory cytokine genes *IL6* and *CXCL8* by cells in HSEs and PBECs in response to SAAP-148 also showed reciprocal changes. IL6 expression significantly reduced following 6 h of SAAP-148 treatment (*P *< 0.02) and 12 h of PBS control (*P* = 0.05) in PBECs, but no such significant changes were determined in HSE cultures (Figure 5e). Expression of *CXCL8* by cells in HSEs was significantly (>1000-fold; *P* < 0.001) increased at 6 h of exposure to SAAP-148 before subsiding to control expression at 24 h, whereas expression of this gene by cells in control PBECs significantly (*P* = 0.031) decreased only after 24 h (Figure 5f). The increased *CAMP*, *DEFB1* and *CXCL8* expression in HSEs correlates with the acute observed antibacterial activity; however, this RNA expression was not maintained across 24 h. Similarly, the observed increase in *DEFB1*, *DEFB4* and *DEFB103A* expression at 24 h in PBECs could indicate contributions by these HDPs to the delayed antibacterial activity observed. Expression of these HDPs and cytokines in response to SAAP-148 is distinct between HSE and PBEC cultures; however, expression in neither culture correlated strictly with the antibacterial activity observed, and their contributions to such activity are likely overshadowed by the direct activity of SAAP-148.

### Antibacterial effects of combinations of SAAP-148 and post-infection exposure to halicin in HSEs and PBECs

Previous studies have established the synergistic interactions between SAAP-148 and halicin against planktonic and biofilm-established bacteria.^14,15^ Here, we further investigated if such interactions persist when combining the pretreatment of HSEs and PBECs with SAAP-148 and post-infection treatment with halicin, as sequential therapy is a preferred strategy to reduce AMR development.^31^ Checkerboard analysis of the two treatments revealed synergistic interactions between the two treatments in HSEs (Table 2). Overall, the treatments acted synergistically against MRSA in CF and CA fractions, with both agents promoting the activity of the other. Interestingly, SAAP-148 pretreatment of PBECs was indifferent in combination with halicin against MRSA in both CF and CA fractions (Table 3). Despite the promotion of halicin’s activity by SAAP-148 (FIC_S→H_ < 0.25), halicin failed to promote the peptide’s activity. These results underscore the distinct effects of pretreatment with SAAP-148 and post-infection treatment with halicin between HSEs and PBECs, emphasizing the potential use of this strategy in skin but not lung tissue.

**Table 2.** Effect of SAAP-148 pretreatment, halicin treatment and combinations thereof on MRSA counts in HSE models

Fraction	Agent	Alone MIC, mg/L	Combined MIC, mg/L	FIC_S→H_	FIC_H→S_	FICi
CF	SAAP-148	168	21	0.13	0.13	0.26
	Halicin	27	3.4			
CA	SAAP-148	168	42	0.13	0.25	0.38
	Halicin	27	3.4			

SAAP-148 (0–335 mg/L) was applied to HSEs 1 h prior to inoculation with MRSA LUH14616, whereas halicin (0–54 mg/L) was provided 1 h after inoculation and maintained for 3 h. When combined, models were exposed to SAAP-148 (0–335 mg/L) for 1 h, washed, then inoculated with MRSA, and 1 h thereafter halicin (0–54 mg/L) was applied for 3 h. The fractional inhibition concentration (FIC_S→H_, FIC_H→S_) and index (FICi) scores are calculated from the MICs of agents alone and combined for cell-free (CF) and cell-adherent (CA) fractions.

**Table 3.** Effect of SAAP-148 pretreatment, halicin treatment and combinations thereof on MRSA counts in PBEC models

Fraction	Agent	Alone MIC, mg/L	Combined MIC, mg/L	FIC_S→H_	FIC_H→S_	FICi
CF	SAAP-148	335	168	0.01	0.50	0.51
	Halicin	54	0.8			
CA	SAAP-148	335	168	0.25	0.50	0.75
	Halicin	54	13.5			

SAAP-148 (0–335 mg/L) was applied to PBECs 1 h prior to inoculation with MRSA LUH14616, whereas halicin (0–54 mg/L) was provided 1 h after inoculation and maintained for 3 h. When combined, models were exposed to SAAP-148 (0–335 mg/L) for 1 h, washed, then inoculated with MRSA, and 1 h thereafter halicin (0–54 mg/L) was applied for 3 h. The fractional inhibition concentration (FIC_S→H_, FIC_H→S_) and index (FICi) scores are calculated from the MICs of agents alone and combined for cell-free (CF) and cell-adherent (CA) fractions.

## Discussion

Here, we have demonstrated that exposure of the HSE and PBEC cultures to SAAP-148 prior to infection prevents colonization by AMR *S. aureus*, with HSEs remaining uncolonized for up to 24 h and SAAP-148 eliminating *S. aureus* at lower concentrations than in the PBEC cultures. Subsequently, it was found that this activity in HSEs is also present against an AMR *P. aeruginosa* strain, consistent with previously observed broad-spectrum antimicrobial activity shown for SAAP-148. Due to the peptide’s greater activity in HSEs than in PBECs, we next demonstrated via confocal microscopy the extensive SAAP-148 uptake and nuclear accumulation in HSEs, a feature absent from treated PBEC cultures. This may suggest that the different protective activities observed were due to the properties of the cultures. LL-37—the peptide from which SAAP-148 is inspired—is endocytosed and accumulates in the nucleus of epithelial cells,^32^ stimulating chemotaxis via downstream phosphorylation of epidermal growth factor receptor (EGFR) and activation of signal transducer and activator of transcription 3 (STAT3).^33^ In the same vein, SAAP-148 is likely trafficked similarly in HSEs, while also associating with cell membranes in the apical layers and forming a chemical barrier to bacterial colonization.

To clarify that the observed activity in HSEs was due to the peptide’s activity and not due to endogenous factors, differences in gene expression between the HSE and PBEC cultures were explored. In HSEs, the expression of the genes for endogenous LL-37 and hBD-1 were transiently increased after 6 h of treatment with SAAP-148, whereas hBD-3 gene expression increased after 12–24 h. In PBECs, the expression of hBD-2 was also transiently increased at 6 h in SAAP-148–treated cells, with an additional peak in expression for both hBD-1 and hBD-2 at 24 h. These expression patterns did not match the protective activities observed in the respective tissue cultures. Similarly, expression of IL-6 and IL-8 cannot be linked to the observed antimicrobial activities in HSEs and PBECs, but indicates how these tissues may stimulate immune responses after SAAP-148 treatment. Finally, our use of SAAP-148 and halicin in sequential treatment—and the synergistic activity observed—highlights the agents’ utility beyond their established activity when applied in combination simultaneously.^14,15^ Further, we established that synergy can be observed between the agents when SAAP-148 is applied before and halicin after infection.

The peptide template for designing SAAP-148, LL-37, has been used previously in the prevention of bacterial adhesion and biofilm formation;^34–36^ however, it has rarely been employed as a prophylactic agent in sequential antibiotic therapy, not least in robust tissue cultures like HSEs and PBECs or *in vivo*. Investigations of endogenous and exogenous LL-37 in PBECs or similar bronchial epithelial cultures largely support its antibacterial activity in such tissues.^37,38^ However, in our study SAAP-148 activity in PBECs was limited in comparison with HSEs. The higher peptide concentrations required, shorter effective window of treatment, and limited activity against *P. aeruginosa* suggest that the chemical properties of SAAP-148 may limit its use as a preventative agent in lung tissue. The minor antibacterial activities noted in PBEC CF fractions after 1 h pretreatment and in CA fractions after 24 h may, respectively, indicate a weak direct antibacterial activity after SAAP-148 application, and potential induction of other host defence factors (i.e. hBD-1). This is further supported by the difference in SAAP-148 localization between the tissue cultures, whereby PBECs did not prominently retain or internalize the peptide. LL-37 is expressed by keratinocytes and stored in lamellar bodies of the stratum corneum to aid barrier function and immunity,^39^ and as SAAP-148 does not retain the native anionic charges of LL-37,^9^ its cationic charge likely contributes to its sequestration and prolonged antibacterial activity in HSEs. It is important to note the differences in cultures used between HSE and PBEC. Whereas the HSE model is based on the use of a single immortalized cell line, the PBEC model used non-transformed primary cells as donor mixes derived from five different donors to generate a mucociliary differentiated epithelial layer.

Other contributory factors to the lower SAAP-148 activity in PBECs could include the mucus secreted by differentiated cells and the ciliary movement, which could interfere with the binding of SAAP-148 to cell membranes. Although the MIC of SAAP-148 for *S. aureus* is sufficiently low as to not induce toxicity in monolayer epithelial cells or differentiated HSEs,^14^ the lack of activity in PBECs could also be attributed to adverse effects in the cultures, such as weakening of intercellular junctions or interactions with culture medium components. The preparation of PBEC cultures for confocal imaging may have also interfered with the retention of SAAP-148 in the cells or their mucous matrix, or the peptide may have become complexed or degraded in such. LL-37 notably interacts with a variety of cell surface receptors to mediate gene expression, including G-protein–coupled receptors, Toll-like receptors, transmembrane channels and receptor tyrosine kinases, such as EGFR.^40^ LL-37 is known to transactivate EGFR and localize to the perinuclear space,^32^ inducing HDP and both IL-6 and IL-8 expression and release from both keratinocytes and bronchial epithelial cultures.^29,41^ SAAP-148 stimulation of HSEs appears to replicate this membrane and perinuclear localization, as well as inducing IL-8 expression within the first hours of exposure, in contradiction to the effects of other synthetic HDP derivatives.^42^ Although these traits are indicative that SAAP-148 preserves the activity of LL-37, its differential activity between HSEs and PBECs is attributable largely to its retention in skin tissue.

Our previous studies have identified the synergistic activity of SAAP-148 and halicin against AMR *Acinetobacter baumannii*, *E. coli*, *K. pneumoniae* and *S. aureus*, in particular their inhibition of biofilm formation and colonization of 3D epithelial models.^14,15^ The extension of this efficacy from a combined application to sequential treatment broadens the potential application of both agents, and supports sequential therapy as a strategy to prevent the further development of bacterial AMR.^5^ Whereas SAAP-148 acts by carpeting bacterial membranes prior to membrane destabilization and lysis,^43^ halicin induces bacterial cell death by disrupting the bacterial proton motive force and sequestering free iron.^13^ As the effect of pretreatment with SAAP-148 alone was poorly effective in PBECs, its promotion of halicin’s activity was surprising and could prove effective under other conditions. However, the overall indifferent interaction with post-infection halicin underscores the need for a broader toolkit of synergistic agents to attend the varied circumstances of AMR bacterial infections. As discussed previously, HSEs are composed of an immortalized keratinocyte cell line from a single donor, whereas PBEC cultures are developed with primary cells derived from several donors, and therefore we cannot exclude that the cell sources are an influence on outcomes, nor the lack of a cellular immune component in such models. Regardless, SAAP-148—and the growing evidence of its activities in new infection models—are welcome additions in the exploration of synergy against AMR bacteria.

Our findings support the use of HDPs in combination and subsequent antibacterial therapies to improve treatment efficacy, decrease the demand for other antibiotics, and directly combat AMR bacterial infections while indirectly minimizing the further development of AMR. These approaches reduce detrimental effects, such as the toxicity observed from SAAP-148 in monolayer culture;^14^ however, functional augmentation can also reduce such effects, as observed with its formulation in nanogels and nanoparticles.^44,45^ Extending the use of SAAP-148 and halicin beyond topical application—and in combination with other non-chemical treatments^46^—could offer means for preventing AMR infection in high-risk environments, for example, nosocomial infection of wound and burn sites, and costly medical interventions due to, for example, prosthetic implant infections.^47,48^ In conclusion, this study highlights the potential of SAAP-148 in the prevention and—in combination with halicin—treatment of AMR infections in epithelial tissues. Its capacity to protect 3D tissue models from bacterial colonization, modulate host immune responses and synergize with subsequent antibiotic treatment—demonstrated here with halicin—underscores its therapeutic potential.

## Supplementary Material

dlaf050_Supplementary_Data

## Supplementary data

Figures S1 and S2 are available as Supplementary data at *JAC-AMR* Online.
