# Supplementary material for: SAAP-148 and halicin exhibit synergistic antimicrobial activity against antimicrobial-resistant bacteria in skin but not airway epithelial culture models
Source: JAC Antimicrob Resist. 2025 Apr 11;7(2):dlaf050. doi: 10.1093/jacamr/dlaf050 (PMC11986330; doi:10.1093/jacamr/dlaf050)
Supplement: dlaf050_Supplementary_Data [file dlaf050_supplementary_data.docx]

**Supplementary Materials**

**Supplementary Figure 1. Quantification of cellular fluorescence in HSEs and ALI-PBECs exposed to FITC-SAAP-148.**

****HSEs (**A**) and ALI-PBEC cultures (**B**) were exposed to either PBS for 1 h or FITC-SAAP-148 for 1, 6, or 24 h and imaged via confocal fluorescence microscopy. Images were taken with 40x oil immersion lens, and cellular fluorescence quantified with CellProfiler (version 4.2.8). Results are expressed as box-and-whisker plots of the Tukey range, with outliers plotted individually. Data are from three inspection fields surveyed per sample, from three independent experiments performed in duplicate. Statistical differences are depicted as * for p ≤ 0.05 as calculated by one-way ANOVA or Kruskal Wallis tests with Dunn’s adjustment for multiple comparisons. NB: The difference in log scale of the y-axis for A and B.

**Supplementary Figure 2. Quantification of nucleated cells in HSEs and ALI-PBECs exposed to FITC-SAAP-148.**

****HSEs (**A**) and ALI-PBEC cultures (**B**) were exposed to either PBS for 1 h or FITC-SAAP-148 for 1, 6, or 24 h, stained with DAPI (nucleus), and imaged via confocal fluorescence microscopy. Images were taken with 40x oil immersion lens, and nucleated cells in models quantitated with CellProfiler (version 4.2.8). Results are expressed as box-and-whisker plots of the Tukey range, with outliers plotted individually. Data are from three inspection fields surveyed per sample, from three independent experiments performed in duplicate. Statistical differences are depicted as * for p ≤ 0.05 as calculated by one-way ANOVA or Kruskal Wallis tests with Dunn’s adjustment for multiple comparisons.
